# Application of TraDIS to define the core essential genome of *Campylobacter jejuni* and *Campylobacter coli*

**DOI:** 10.1186/s12866-023-02835-8

**Published:** 2023-04-06

**Authors:** Emily Stoakes, Keith Turner, Dave J. Baker, Maria Suau Sans, Muhammad Yasir, Lajos Kalmar, Ruby Costigan, Martin Lott, Andrew J. Grant

**Affiliations:** 1 Department of Veterinary Medicine, University of Cambridge, Madingley Road, Cambridge, UK; 2 Quadram Institute Bioscience, Norwich Research Park, Norwich, UK; 3 MRC Toxicology Unit, University of Cambridge, Tennis Court Road, Cambridge, UK

**Keywords:** Campylobacter spp, Campylobacter jejuni, Campylobacter coli, TraDIS, Transposon mutagenesis, Essential genes

## Abstract

*Campylobacter* species are the major cause of bacterial gastroenteritis. As there is no effective vaccine, combined with the rapid increase in antimicrobial resistant strains, there is a need to identify new targets for intervention. Essential genes are those that are necessary for growth and/or survival, making these attractive targets. In this study, comprehensive transposon mutant libraries were created in six *C. jejuni* strains, four *C. coli* strains and one *C. lari* and *C. hyointestinalis* strain, allowing for those genes that cannot tolerate a transposon insertion being called as essential. Comparison of essential gene lists using core genome analysis can highlight those genes which are common across multiple strains and/or species. Comparison of *C. jejuni* and *C. coli,* the two species that cause the most disease, identified 316 essential genes. Genes of interest highlighted members of the purine pathway being essential for *C. jejuni* whilst also finding that a functional potassium uptake system is essential*.* Protein–protein interaction networks using these essential gene lists also highlighted proteins in the purine pathway being major ‘hub’ proteins which have a large number of interactors across the network. When adding in two more species (*C. lari* and *C. hyointestinalis)* the essential gene list reduces to 261. Within these 261 essential genes, there are many genes that have been found to be essential in other bacteria. These include *htrB* and *PEB4*, which have previously been found as core virulence genes across *Campylobacter* species in other studies. There were 21 genes which have no known function with eight of these being associated with the membrane. These surface-associated essential genes may provide attractive targets. The essential gene lists presented will help to prioritise targets for the development of novel therapeutic and preventative interventions.

**Supplementary Information:**

The online version contains supplementary material available at 10.1186/s12866-023-02835-8.

## Introduction

*Campylobacter* species are the leading cause of bacterial gastroenteritis worldwide, costing the UK ~ £0.71 billion per annum [1]. In the UK, about 90% of campylobacteriosis cases are caused by *Campylobacter jejuni*, whilst *Campylobacter coli* causes about 10% [1]. However, there are other ‘emerging’ species such as *Campylobacter lari* and *Campylobacter hyointestinalis* that have been reported sporadically from human cases and can cause extraintestinal infections [2–4]. The major contributor to *Campylobacter*-associated human disease is the consumption of contaminated meat products but other sources include contaminated water [5]. In the UK, over 55% of *C. jejuni* cases have been associated with poultry [6]. *C. coli* is linked to chickens, but is considered the dominant species that colonises, and sometimes causes infection in, pigs. Conversely, in some countries including France, *C. coli* cases have been associated more often with cattle [7, 8].

Human campylobacteriosis usually presents as diarrhoea, however, clinical manifestations can also include (but are not limited to) long-term complications such as reactive arthritis and Guillain-Barré syndrome. The cost of campylobacteriosis in low and middle-income countries remains unknown, but it is believed the burden of *Campylobacter* diarrhoea in children living in these regions has been greatly underestimated [9]. No vaccines exist against campylobacteriosis for humans nor for other animals, and whilst antibiotics can treat illness, they cannot prevent it effectively and may have the undesirable consequence of promoting resistance [10]. Worldwide there has been a rapid increase in antibiotic resistant *Campylobacter* strains, making them a serious and growing public health threat [11].

Several studies have described the pangenomes (*i.e*., all of the genes that exist within a species) of *C. jejuni* and *C. coli* and their population structures. *C. jejuni* strains form a weak clonal complex structure due to its host switching and more generalist lifestyle, whereas *C. coli* forms three distinct clades; with Clade I associated with agriculture, whilst Clades II and III are associated with environmental sources [12–14]. The complete and partial genome sequences available for different *Campylobacte*r species enable pangenome bioinformatic analyses to define the core genes, present across all or most strains, and shell genes, present in more than two strains. However, the genome sequences in themselves do not identify which genes are likely to be essential for survival and growth.

Essential genes, defined as genes indispensable for growth and/or survival, of bacterial pathogens, may encode components of novel biochemical pathways or potential targets for antibacterial drug development [15–17]. Essential genes are attractive targets for the development of novel antimicrobials as the development of compounds that inhibit the functions encoded by these genes are more likely to have antimicrobial activity than strategies targeting genes that encode non-essential functions [18]. Gene essentiality can be assessed by targeted gene disruptions, where genes that cannot be disrupted are defined as being essential [16, 17]. This type of approach is both labour and time consuming. Instead, developments in next generation sequencing (NGS) have facilitated Transposon Insertion Sequencing (TIS, *e.g.,* TnSeq, InSeq, TraDIS) to find candidate essential genes in a genome-wide manner [15, 16]. There is a requirement to understand essential genes and their cellular functions in pathogenic bacteria to appreciate how bacterial growth is controlled, and to accelerate the development of novel antimicrobial strategies [16, 19].

Transposon (Tn) gene inactivation libraries have previously been created in well characterized *C. jejuni* strains such as 11168, M1cam and 81–176 [20–23], with smaller Tn mutant libraries also created in less-studied strains such as *C. jejuni* 01/51 [24]. Comprehensive Tn inactivation libraries can be used to identify genes, or regions within genes, that cannot tolerate Tn insertions and thus indicate genes, or parts of genes, that are likely to be essential for the fitness/survival of the bacterium.

In this study, we have developed TraDIS for *Campylobacter* species and used this to generate comprehensive Tn libraries in *C. jejuni, C. coli, C. lari and C. hyointestinalis*. Pangenome comparisons between strains and species were used to define the essential genes in these four species and core essential genes across these species. In addition, we used informatic approaches to predict protein–protein interaction networks to identify core genes, pathways, and proteins with multiple interaction partners. This study aimed to identify strain-independent targets across *Campylobacter*. These targets may enable interventions that result in the decrease of the bacterium and illness across both animals and humans.

## Materials and Methods

### Bacterial strains and growth conditions

*Campylobacter* species and strains used in this study and their genotypes are summarized in Table 1. All *Campylobacter* species and strains were cultured in Mueller–Hinton (MH) broth or on MH agar, with the addition of 10 μg/ml chloramphenicol when required for selection. Liquid cultures were grown shaking at 200 rpm. Strains were grown at 42 °C under microaerophilic conditions of 5% O_2_, 5% CO_2_, 5% H_2_, 85% N_2_ in an M95 variable atmosphere workstation (Don Whitley). Bacterial stocks were stored at -80 °C in MicroBank vials (ProLabs). All strains were grown directly from freezer stocks onto MH agar plates for 48 h, and then re-streaked for a further 16 h before use.

**Table 1:** Bacterial strains and plasmids used in the study

Strain or Plasmid	Relevant genotype or description	Reference
***C. jejuni* strains**		
M1cam	Wild-type; derivative of M1	[25, 26]
11168	Wild-type	[27]
81–176	Wild-type	[28]
80512	Wild-type	[29]
80864	Wild-type	[29]
50520408	Wild-type	[29]
***C. coli* strains**		
15–537360	Wild-type	[30]
H062180535	Wild-type	[31]
H102680185	Wild-type	[31]
CCN182	Wild-type	[31]
***C. hyointestinalis***		
ATCC35217	Wild-type	[32]
***C. lari***		
ATCC35221	Wild-type	[33]
***E. coli* strains**		
NEB 5α	F^–^φ80*lac*ZΔM15 Δ(*lac*ZYA-*arg*F)U169 *rec*A1 *end*A1 *hsd*R17(r_K_^–^,m_K_^+^) *pho*A *sup*E44 λ^–^ *thi*-1 *gyr*A96 *rel*A1	New England Biolabs
BL21 pLysS	F^−^*ompT hsdS*_B_(r_B_^−^ m_B_^−^) *gal dcm* (DE3) pLysS (Cam^R^)	Novagen
**Plasmids**		
pRC1-*mariner*	*mariner* Tn (Cm^R^) donor plasmid used for in vitro Tn mutagenesis; pRC1 backbone (pSV009 derivative), Amp^R^	[23]
pMalC9	Plasmid for expression of *Himar1* transposase fused to a maltose binding protein, Amp^R^	[34]

*Escherichia coli* strains, for propagation of plasmids or expression of the *Himar* transposase, were routinely grown in Lysogeny broth (LB) or on LB agar with the addition of 100 μg/ml ampicillin when required for selection. All strains were grown at 37 °C, with 200 rpm agitation when in liquid broth.

### *Campylobacter* genome nucleotide sequence determination

To determine the nucleotide sequences of the *C. jejuni* 80512, 80864, 50520408, *C. coli* H062180535, H102680185, CCN182, *C. lari* 35221 and *C. hyointestinalis* 35217 genomes, genomic DNA was extracted using a DNeasy Blood and Tissue kit (Qiagen). After adjustment of genomic DNA to 5 ng/µl, 2 µl was mixed with 5 μl of a pre-made tagmentation mix (0.5 µl of TB1 Tagment DNA Buffer was mixed with 0.5 µl BLT, Tagment DNA Enzyme (Illumina) and 4 µl PCR grade water) and heated to 55 °C for 15 min. PCR was then performed using a master mix of 4 µl KAPA2G buffer, 0.4 µl dNTPs, 0.08 µl Polymerase and 4.52 µl PCR grade water which was added to the tagmented DNA. To each sample, 2 μl of each P7 and P5 of Nextera XT index kit v2 primers was added. The PCR conditions were 72 °C for 3 min, 95 °C for 1 min, 14 cycles of 95 °C for 10 s, 55 °C for 20 s and 72 °C for 3 min. Following the PCR, the libraries were quantified using the Promega QuantiFluor dsDNA System (Promega) and run on a GloMax Discover Microplate Reader (Promega). Libraries were pooled following quantification in equal quantities. The final pool was double-SPRI size-selected between 0.5 and 0.7X bead volumes using KAPA Pure Beads (Roche). The final pool of PCR amplified DNA was quantified using a Qubit 3.0 fluorometer (Invitrogen) and the molarity determined using a Tapestation 4200 system with a D5000 ScreenTape (Agilent). The DNA pool was loaded at a final concentration of 1.5 pM on an Illumina Nextseq 500 sequencer using a Mid Output Flowcell to generate nucleotide sequence reads.

A custom software pipeline was used to assemble the sequence reads into whole genome sequences. Briefly, Shovill v1.0.04, including SpaDES v3 13.0 was used for assembly and Bakta v1.6.1 was used for annotation using Database v4.0 [35].

### Core genome analysis within species

For previously sequenced strains—*C. jejuni* M1cam (GenBank accession number: CP012149.1), 11168 (GenBank accession number: NC_002163.1), 81–176 (GenBank accession number: CP000538.1) and *C. coli* 15–537360 (GenBank accession number: CP006703.1)—the curated genomes were used for this analysis. Both the curated genomes and strains annotated by Bakta meant that (at least some) pseudogenes were annotated as pseudogenes and therefore not taken into account in the core genome and were not re-annotated by Prokka within Panaroo. The core genome (genes that were in common across 100% of the strains in this study) were identified using Panaroo with an identity threshold of 95% [36]. A further filtering step was used to remove gene clusters that Panaroo flagged as potential pseudogenes, genes with unusual lengths, fragmented genes that included multiple sequence IDs (locus tags) or genes that had been ‘refound’ by the software. Using both an identity threshold of 95% and a filtering step means that the core genome reported here is more stringent than necessary and might miss certain genes that may belong in the core list. For example, *flaA* and *flaB,* in *Campylobacter jejuni* M1cam and 81–176 were removed in in this filtering due to the identity between them being above 95%. Although these genes will be removed from the core genome, this filtering step also will remove gene clusters that collapse into gene families that are fragments of pseudogenes annotated as functional genes. Previous analysis using all genomes reannotated using a different annotation software in combination with Panaroo led to gene clusters being called in the core genome which were pseudogenes or genes being ‘refound’ but were not annotated in the well annotated and studied strains such as *C. jejuni* 11168, 81–176 and M1cam. For example, the potassium transporting ATPase *kdpABC* was called as core, despite the *kdpA* gene being a pseudogene in *C. jejuni* 11168. Four smaller genes were predicted in *C. jejuni* 11168 that collapsed into one gene family in Panaroo. Removing groups of genes with more than one sequence ID associated, ensures that potential fragmented genes which are not genes are not considered and ensures that the core genome list, although smaller, includes only actual genes. Both core gene lists and lists after filtering can be found in Table S4.

### Core genome analysis across species

We formed clusters of homologous genes in a two-step process. First, we searched for high-level protein similarity in pairwise comparisons by using all combinations between gene-sets from different species, using protein BLAST [37]. In the second step, communities (clusters of homologous genes) were formed by using each gene as a node and homology hit information (e-value < 1e-25) as edges in a network model. Final communities were defined by using the Perl programming language implementation of the Louvain network resolution method (SNA-Network-0.21 CPAN package, https://metacpan.org/dist/SNA-Network). The final list of gene clusters allowed us to define pan-specific conserved genes and between-species gene conservations (Table S5).

### Construction of *mariner *Tn mutant libraries in *Campylobacter* strains

The *mariner* Tn was custom-synthesized by GeneArt (ThermoFisher Scientific) and incorporates the *mariner* mosaic ends flanking the *Campylobacter* chloramphenicol acetyltransferase resistance gene (*cat*) from pAJG39 including its promoter and putative terminator [38]. The *mariner* DNA fragment also included nucleotide sequences near one end for binding of a custom-made Illumina i5 sequencing primer allowing the generation of nucleotide sequence reads initiated from *mariner* and proceeding into the adjacent insertion sites. The *mariner* DNA fragment was incorporated into plasmid vector pRC1 (a derivative of pSV009 [26]) resulting in pRC1-*mariner*.pRC1-*mariner* was propagated in *E. coli* DH5α using conditions described above and plasmid DNA was isolated using an NEB miniprep kit, as per the manufacturer’s instructions. *Campylobacter* genomic DNA was purified using Genomic-Tip Columns (Qiagen), according to the manufacturer’s instructions.

For Tn mutagenesis, the *Himar* transposase was expressed from pMalC9 and purified as described in Akerley et al. [39]*.* For in vitro transposition reactions, 2 μg of *Campylobacter* genomic DNA was incubated with 1 μg of pRC1-*mariner* and the *himar* transposase in transposition buffer (40% glycerol, 80 μM DTT, 100 mM HEPES, pH 7.9, 1 mg/ml BSA, 400 mM NaCl_2_, 20 mM MgCl_2_). Individual 20 μl aliquots of the reaction were incubated for 4 h at 30 °C. The aliquots of Tn-mutated DNA were then combined and purified by ethanol precipitation. The DNA was then dissolved in water and end-repaired with T4 Ligase and T4 DNA polymerase (NEB). The repaired transposed DNA was purified by ethanol precipitation and resuspended in water. Genetic transformation of the *Campylobacter* strains was performed with 20 μl of the Tn mutated DNA using natural competence, as described previously [40]. For each Tn mutant library made in this study, 12 transformations were performed, each requiring one in vitro transposition reaction. DNA-treated bacterial cells were combined across all transformations and 100 μl spread onto each of 60 MH agar plates containing chloramphenicol at 10 µg/ml. After 2 days of incubation at 42 °C, all the transformants were harvested from each plate using 1 ml of MH broth and the resulting cell suspensions were combined. The resulting Tn mutant library pool was used to inoculate 100 ml of MH broth with 10 μg/ml chloramphenicol at a starting OD_600nm_ of 0.1 and grown overnight at 200 rpm. Aliquots of 1 ml for each Tn library were frozen at -80 °C using MicroBank tubes (ProLabs).

### Generation and analysis of TraDIS nucleotide sequence reads

Genomic DNA from each Tn mutant library was extracted as described above. Genomic DNA (25 ng) was fragmented and ligated to Illumina sequencing adapters using the Nextera XT tagmentation kit (Illumina). For this, 1 µl genomic DNA was mixed with 24 µl of tagmentation mix (12.5 µl of TD Tagment DNA Buffer (Illumina) was mixed with 2.5 µl TDE1, Tagment DNA Enzyme (Illumina) and 14 µl PCR grade water) and heated to 55 °C for 10 min. PCRs were then performed by adding 21 µl KAPA2G Fast HS Master Mix (Sigma) with 2 µl of each P7 index oligonucleotide (Nextera XT Index Kit v2, Illumina). Customised P5 index oligonucleotides incorporating nucleotide sequences at their 3’-ends, which hybridise with the known sequences near the end of the *mariner* Tn, were also added to each sample (Table S2) to act as PCR primers and the reactions were incubated using the following thermal cycle conditions: 72 °C for 3 min, 95 °C for 3 min, 28 cycles of 95 °C for 10 s, 55 °C for 20 s and 72 °C for 1 min. The resulting PCR products generated from the *mariner* ends include adjacent genomic DNA and these were then quantified using the Quant-IT dsDNA HS assay kit (Invitrogen) and the resulting fluorescence was measured using a FLUOstar Optima microplate reader (BMG Labtech). Following quantification, PCR products from the different reactions were pooled and double-SPRI size-selected using between 0.5 and 0.7X volume of KAPA Pure Beads (Roche). The size-selected pool was quantified using a Qubit 3.0 fluorometer (Invitrogen) and the molarity measured using a Tapestation 4200 with a D5000 ScreenTape (Agilent). The nucleotide sequences of the PCR products were generated using a NextSeq 500 sequencer with a 500/550 high output V2 kit (75 cycles) (Illumina).

### Matching of TraDIS nucleotide sequence reads to the *Campylobacter* reference genome nucleotide sequences

The fastq files written contain the nucleotide sequence reads of the PCR fragments incorporating the known *mariner* end and adjacent genomic nucleotide sequences. These fastq files were processed bioinformatically. For this, reads that did not contain the *mariner* Tn-end sequence (‘GGG(16 bases)GTT’) were first removed using a custom Perl script. Then, reads of less than 40 bases were removed using Cutadapt [41]. The trimmed and filtered reads were matched with the nucleotide sequences of the relevant reference *Campylobacter* genome using the Bio-Tradis software with Smalt as the default mapper [42]. This generated plot files which included the precise location and number of sequence reads that matched the reference genome. Since the reads were generated from the *mariner* Tn insertions, the plot files indicated the precise locations and approximate numbers of all the mutations in the Tn mutant library pool, thereby providing an accurate measure to the composition of the Tn mutant library. The tradis_essentiality.R script from Bio-Tradis was then used which compares the plot file information with the genome annotations to determine the relative number of Tn mutants for each gene. Genes with few or no Tn mutants are likely to be essential, whilst for most genes there are many Tn mutants. The tradis_essentiality.R script also outputs gamma-fitted distribution graphs based on the number of Tn mutants per gene length (Figure S1). this provides a ‘threshold number’ to determine if a gene is to be called as essential [42, 43]. These graphs generally show a bimodal distribution with one mode at or close to *x* = 0, representing the essential genes that generally lack Tn insertions and a second peak further along the *x*-axis representing the non-essential genes that contain numerous Tn insertions. The essential threshold number was used for all libraries. Full essential gene lists for each strain can be found in Table S3.

### Sorting genes into clusters of orthologous groups

Genes were assigned to clusters of orthologous groups (COGs) using Eggnog mapper [44, 45]. To investigate whether any COGs in each genome were enriched in the essential gene list, a Fisher's exact test (*P* < 0.05 or less) was performed within GraphPad Prism. Genes that belonged to multiple COG categories were allocated to all relevant groups.

### Protein interactome analysis

The list of core essential genes was used to construct a protein–protein interaction (PPI) network using STRING (Search Tool for Retrieval of Interacting Genes/Proteins Database) [46]. STRING produces protein–protein interaction (PPI) networks by searching for direction interactions using curated data from text mining, experiments, databases, co-expression, neighbourhood, gene fusion and co-occurance [46]. For the *C.* *jejuni* core essential gene list, locus tags from *C.* *jejuni* 11168 were used as the representative strain. For the *C. coli* core essential gene list, no *C. coli* strains used in this study were present in the STRING database. The core essential list was compared to the *C. coli* NCTC11366 to find homologs (using Panaroo as described above) and the STRING *C. coli* NCTC11366 database was used to create a PPI network. The PPI networks produced by STRING were transferred into Cytoscape (v3.9.1) and default parameters used to analyse the PPI network [47].

To identify hub proteins, methods described by Gollapalli et al*.* were used [48]. Cytohubba was used for further topological analysis of the network [49]. Cytohubba provides 11 different methods to find the top ten proteins within networks. We used it to identify hub proteins by five different classification methods of centrality – degree, betweenness centrality, closeness centrality, bottleneck and radiality. Degree centrality ranks proteins on the number of interactions they have with other proteins (or nodes). The nodes with higher numbers of degrees (or interactions) can be hub proteins as they are usually located at the centre of the network [50–52]. Betweenness centrality ranks proteins on the shortest path passing through proteins (or nodes) within a network [50–52]. Closeness centrality ranks proteins by calculating the average distance of all the shortest paths between a protein and all other proteins [50–52]. Radiality centrality ranks proteins on the diameter of the network. A larger radiality shows that a protein is closer to other proteins, whereas a protein with a small radiality shows that the protein is on the edge of the network [51]. For the Bottleneck method, those proteins with the shortest path are ranked [50–52]. Proteins predicted to be hub proteins using these five methods were then studied for overlaps.

## Results and discussion

### *Campylobacter* strain selection and Tn mutagenesis

Forty-four strains of *C. jejuni* (including three ‘lab’ strains) and thirty of *C. coli* were obtained (Table S1) and each tested in duplicate to identify which had the highest Tn mutagenesis mutation frequency. From this triage process, three ‘lab’ strains of *C. jejuni*—M1cam, 81–176 and 11168, and three clinical strains, 80512, 80864 and 50520408 were selected. Phylogenetically, 11168 (ST(Sequence Type)-45) and 80864 (ST-19) belong to Clonal Complex (CC) ST-21 whereas M1cam (ST-137) and 50520408 (ST-45) belong to CC ST-45. 81–176 (ST-604) belongs to CC ST-42 and 80512 (ST-574) belongs to CC ST-574 [53]. The *C. coli* stains selected were 15–537360, isolated from a human clinical case, H102680185 and H062180535 isolated from chicken meat, and CCN182 isolated from a farm environment. Phylogenetically, these strains fall within Clade 1, with H062180535 being in cluster C of Clade 1, and the other strains in cluster A [31]. The strains 15–537360 (ST-855), CCN182 (ST-827) and H062180535 (ST-827) all belong to CC ST-828, whereas H102680185 (ST-3311) is not linked to a clonal complex [53]. In addition, a strain for each of two ‘emerging’ or ‘other’ *Campylobacter* species, *C. lari* and* C. hyointestinalis* were also selected and Tn mutant libraries created. *C. lari* 35221 was isolated from a seagull, whilst *C. hyointestinalis* 35217 was isolated from the intestines of a pig [32, 33]. The genomes ranged in size between 1.49 and 1.73 Mb with two strains (*C. jejuni* 81–176 and *C. coli* 15–537360) containing plasmids (Table 2).

**Table 2:** *Campylobacter* Tn libraries created in this study. The table includes the species and strain name, the number of unique Tn insertions for each library, the density of Tn insertions for each Tn library (genome sequence length divided by the number of unique Tn insertions) and the number of essential genes called by the Bio-Tradis pipeline [42]

Species	Strain	Size of genome (Mb)	Number of coding sequences	Number of unique Tn insertions	Density of library (Tn insertion per bp)	Number of essential genes
***C. jejuni***	11168	1.64	1668	79,301	21	522
	81–176	1.62	1655	60,672	27	546
	M1cam	1.62	1658	51,213	32	467
	80512	1.7	1779	72,455	24	653
	80864	1.62	1664	97,150	17	534
	50520408	1.69	1725	84,917	20	521
***C. coli***	15–537360	1.66	1666	36,556	45	484
	CCN182	1.73	1788	104,979	17	681
	H062180535	1.68	1739	114,409	15	595
	H102680185	1.72	1744	38,000	45	669
***C. lari***	35221	1.49	1534	98,705	15	563
***C. hyointestinalis***	35217	1.73	1778	49,675	35	562

Between the Tn mutant libraries of each selected strain, the number of unique Tn insertions (*i.e*., different Tn mutants) ranged from 36,556 for strain 15–537360 to 114,409 for strain H062180535, providing, on average, Tn insertions across each genome at least every 45 bp and up to every 15 bp (Table 2). The number of essential genes for the *C. jejuni* strains ranged between 467 genes for *C. jejuni* M1cam to 653 genes for *C. jejuni* 80512, whilst for *C. coli* the number of essential genes ranged between 484 genes for *C. coli* 15–537360 to 669 genes for *C. coli* H102680185 (Table 2). Both *C. jejuni* 81–176 and *C. coli* 15–537360 contain plasmids, however due to the nature of plasmids having individual replication systems and different copy numbers, and being difficult to screen due to this, we have not included analysis of these here [54]. For *C. lari* 35221 and *C. hyointestinalis* 35217, 563 and 562 genes respectively were called as essential. Full essential gene lists can be found in Table S3.

### Functional overview of essential genes within species

**Fig. 1:**
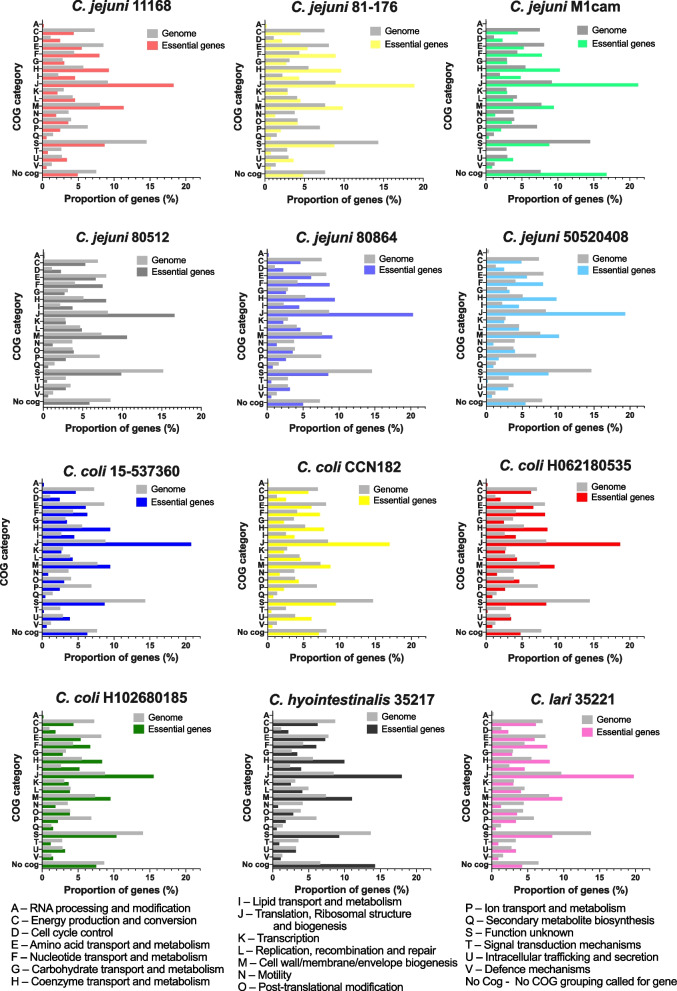
Proportion of genes in COGs sorted from each whole genome compared to the proportion of genes in COGs sorted from the essential genes for each genome. For all strains used in the study, the genes of the genome were sorted into COGs and the number of genes in each COG is expressed as a proportion of the total number of genes in the genome. Similarly, the essential genes were sorted into COGs and the number of genes in each COG is expressed as a percentage of the total number of essential genes. Thus, for example, the essential genes consist of a greater proportion of those coding for functions related to “translation” than the proportion of “translation”-related genes in the whole genome. Some genes are in more than one COG and were allocated into all relevant categories

**Fig. 2:**
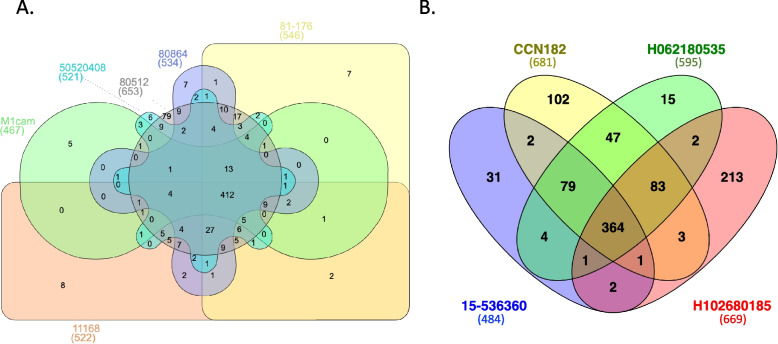
Overlap of essential genes across *C. jejuni* strains (**A**) and *C. coli* strains (**B**). Venn diagrams showing, and comparing, the essential genes for (**A**) . *C.* *jejuni *11168, 81–176, M1cam, 80864, 50520408, and 80512. (**B**) *C. coli* 15–537360, CCN182, H062180535 and H102680185. Numbers within parentheses indicate the number of essential genes for each strain

A functional overview of each genome compared to the essential genes for that strain using Clusters of Orthologous Groups (COG) [55], was performed to investigate any over- or under-representation of COGs for the essential genes (Figs. 1 and 2). Significance of the representation of each group in the essential genes was determined using Fisher's exact test. For all *C. jejuni* strains, there was significant over-representation for several COG categories (Fig. 1, Table 3). These included genes involved in cell cycle control and cell division (D), nucleotide transport and metabolism (F), coenzyme transport and metabolism (H), lipid transport and metabolism (I) and translation (J). There was also significant under-representation across all *C. jejuni* strains for genes involved in cell motility (N), inorganic ion transport and metabolism (P), genes with unknown function (S) and signal transduction mechanisms (T). There are notable strain differences where sets of functional genes are over- or under-represented in some strains but not in others. For example, genes belonging to energy production and metabolism (C) are significantly under-represented in all strains except for *C. jejuni* 80512, whereas essential genes encoding proteins involved in cell wall and envelope biogenesis (M) are significantly over-represented in four out of the six strains. Several functional groups did not have a significant over- or under-representation for essential genes. These included RNA processing and modification (A), carbohydrate transport and metabolism (G), transcription (K), replication and repair (L) and posttranslational modifications (O), intracellular trafficking (U) and defence mechanisms (V).

**Table 3:**
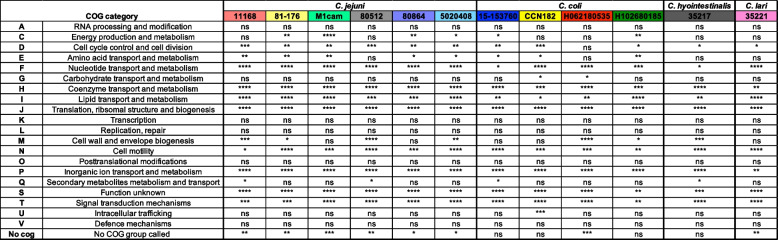
Functional groupings of essential genes which are significantly represented in *Campylobacter* strains and species. Table shows the COG letter code and description and if the COG is significantly over-represented within essential genes compared to the whole genome for each strain tested in this study. Significance was called using Fisher's exact test with a p-value of less than 0.05. ns denotes not significant, * denotes *P* ≤ 0.05, ** denotes *P* ≤ 0.01, *** denotes *P* ≤ 0.001, **** denotes *P* ≤ 0.0001

COGs of the essential genes across the four *C. coli* strains showed a more varied pattern of those categories of genes that were under- and over-represented between strains. Four functional groups were over-represented across the *C. coli* strains: nucleotide transport and metabolism (F), coenzyme transport and metabolism (H) and translation (J), lipid transport and metabolism (I). Cell motility (N), inorganic ion transport and metabolism (P), signal transduction mechanisms (T) and the unknown function category (S) were significantly under-represented. Variations across strains included energy production and metabolism (C) being under-represented in two strains (*C. coli* 15–537360 and H102680185), whereas intracellular trafficking (U) was only significantly over-represented in *C. coli* CCN182. Some functional groups did not have a significant over- or under-representation in any of the four *C. coli* strains; these were RNA processing (A), replication and repair (L), transcription (K), posttranslational modifications (O), and defense mechanisms (V).

Analysis of enrichment of functional groups (Fig. 1) for *C. hyointestinalis* 35217 and *C. lari* 35221 indicates that both species have a significant over-representation of essential genes in cell cycle control (D), nucleotide transport and metabolism (F), coenzyme transport and metabolism (H), lipid transport and metabolism (I), translation (J) and a significant under-representation of essential genes in cell motility (N), ion transport and metabolism (P), signal transduction mechanisms (T) and genes of unknown function (S). *C. hyointestinalis* 35217 also had a significant over-representation of essential genes in cell wall and envelope biogenesis (M).

Across all *Campylobacter* species and their strains tested, COGs representing machinery for translation (J), coenzyme transport and metabolism (H) and nucleotide transport and metabolism (F) are significantly over-represented in essential genes (Table 3). These over-represented groups indicate that these processes are necessary for growth and survival and are consistent with previous studies employing Tn mutagenesis to evaluate essential genes of *C. jejuni* and other bacteria such as *Pseudomonas protegens* [23, 56]*.* It is predictable that many genes coding for translation functions will be essential, as an inability to synthesise proteins would be detrimental to growth. This is true in other studies in *E. coli* where many ribosomal proteins are essential [57]. Previous studies also found cell cycle control (D) to be over-represented by essential genes [23, 56]. Across the *Campylobacter* species and strains used in our study, our data also confirmed this observation, except for *C. coli* H062180535. Inorganic ion transport and metabolism (P) and cell motility (N) were significantly under-represented across all Tn libraries. Motility is integral to the infectious lifestyle of *Campylobacter* (at least confirmed in *C. jejuni*) and is essential for host colonization [58]. However, chemotaxis and motility functions are unlikely to be required for growth in the complex nutrient-rich medium used for this study. Previous Tn mutant libraries produced and selected using different conditions, for *C. jejuni* M1cam, 81–716 and 11168 showed that motility was neither significantly over- nor under-represented [23]. The use of rich media also explains the significant under-representation of inorganic ion transport and metabolism as these pathways may not be required for growth, an observation again consistent with other Tn mutagenesis studies of bacteria [59].

### Defining essential genes within different *Campylobacter* species

In previous studies, analysis of essential genes within *Campylobacter* has focused on one strain alone or a few lab-adapted strains and studies have been reported only with *C. jejuni* [20, 21, 23, 24]. Combining both core genome analysis and the essential gene lists from the TraDIS library analysis enabled us to determine core essential genes across the species tested under the conditions for which the Tn mutants were produced which in this study was microaerophilic conditions (5% H_2_, 5% CO_2_, 5% O_2_, 85% N_2_) at 42 °C using Mueller Hinton agar (Table 4).

**Table 4:**
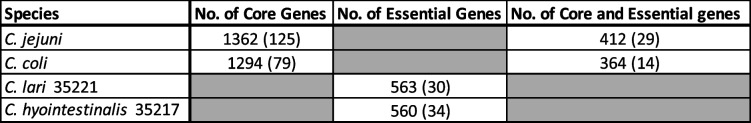
Number of shared genes and essential genes that are in all strains for each *Campylobacter* species. Table shows the number of core genes called by Panaroo for the six *C. jejuni* strains and the four *C. coli* strains, with the number of hypothetical genes in parentheses. For *C. lari* 35221 and *C. hyointestinalis* 35217, as there is only one strain for each species, it is not possible to define the core genes for each species. The number of essential genes is also shown, with the number of hypothetical genes in parentheses

To define the core genome of the six *C. jejuni* strains and separately for the four *C. coli* strains, Panaroo was used [36]. In this study, genes had to be present in all strains to be called as core. Further filtering to remove potential pseudogenes, fragmented genes or genes with unusual length was used to further define the core genome list. (Table S4). There were 1362 core genes across the *C. jejuni* strains and 1294 core genes across the *C. coli* strains. For *C. jejuni* and *C. coli*, within the core genome, approximately 9% and 6% of genes respectively are annotated as encoding hypothetical proteins only, with no other predicted annotation (Table 4).

For *C. jejuni*, there were 412 essential genes in common between all strains (Fig. 2A). For each strain, most of the essential genes (between ~ 95.5%-98.5%) were also common across all six strains. The largest difference was in *C. jejuni* 80512, where 1.07% (7 out of 653**)** of the essential genes did not exist in any other strain tested in our study. As the only genome from the ST-574 complex this may be expected due to potential differences in gene presence/absence within the strain.

For *C. coli*, 364 essential genes were common between all strains tested (Fig. 2B). Unlike *C. jejuni*, *C. coli* showed a greater range in the number of essential genes that did not form part of the core genome (~ 70%-98.5%). For example, with *C. coli* H102680185 28.5% (191 out of 669) of the essential genes did not have a homolog in any other *C. coli* strain used in this study. Similar to the *C. jejuni* 80512 strain, *C. coli* H102680185 belongs to a different ST complex than the other strains included in this study.

### Comparison of core essential genes across species

**Fig. 3:**
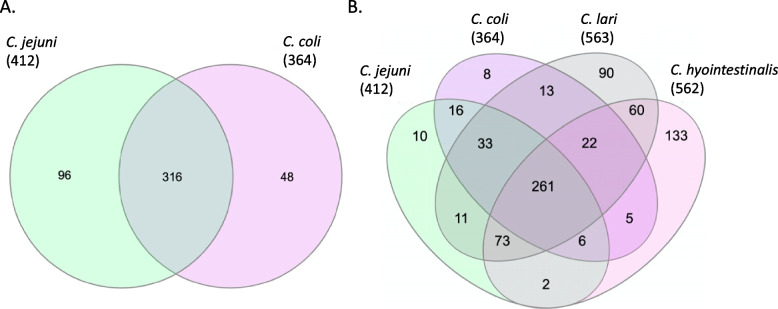
Overlap of essential genes between *C. jejuni* and *C. coli* (**A**)*,* and between all species tested in this study (**B**). Venn diagrams showing, and comparing, the number of core essential genes of the six *C. jejuni* strains and the four *C. coli* strains (**A**), and core essential genes of the six *C. jejuni* strains and the four *C. coli* strains and the essential genes from *C. lari* 35221 and *C. hyointestinalis* 35217 (**B**)

Using the essential genes from multiple strains and species, we reasoned that it would be possible to highlight proteins and pathways that could be targeted for species-level control, or indeed for cross-species intervention. Using an in-house pipeline, we were able to compare the core essential genes between *C. jejuni* and *C. coli* (Fig. 3, Table 5). This is of particular interest as most of the disease burden is attributed to these two species. In theory, by comparing multiple strains within each species and then comparing these species to each other, essential genes may be found as potential targets for intervention either for each species alone or together. The addition of the two other species in this study, although not (so far) as important to human disease cases and representing only one strain each, permits a further investigation across more *Campylobacter* species for potential targets for intervention.

**Table 5:**
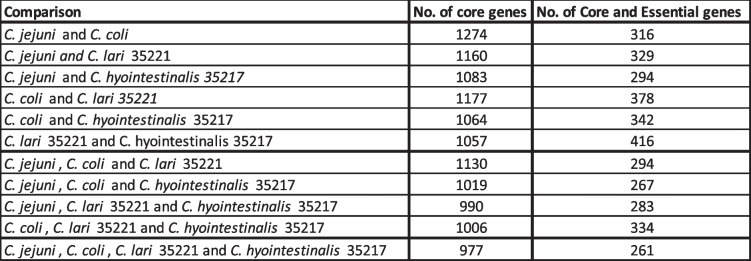
Intra-species comparisons of core and essential genes. The table shows two-species, three-species, or four-species comparisons. The number of core genes called through our custom in-house pipeline is shown (Table S5), alongside the number of genes that are core and essential within that comparison

**Fig. 4:**
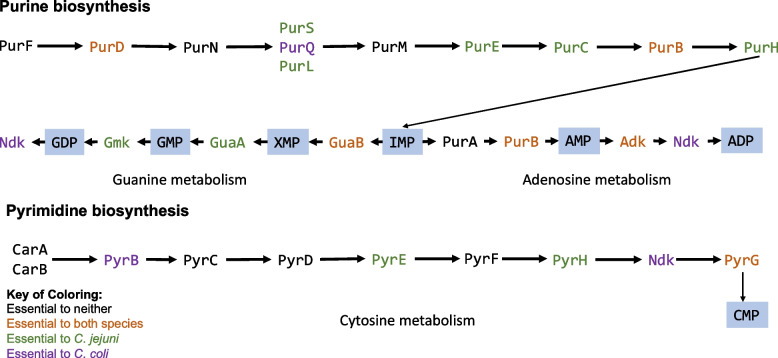
Essential proteins within the purine and pyrimidine biosynthesis pathway. The pathways show the proteins involved in purine and pyrimidine biosynthesis with the products of the pathways in blue filled boxes. All proteins are part of the *C. jejuni* and *C. coli* pathways and exist in both species, however, differ in essentiality. Proteins in brown are essential to both species, proteins in green are those only essential to *C. jejuni,* whilst proteins in purple are only essential to *C. coli*. Abbreviations: GDP-Guanosine diphosphate, GMP-Guanosine monophosphate, XMP-Xanthosine monophosphate, IMP-Inosine monophosphate, AMP-Adenosine monophosphate, ADP-Adenosine diphosphate. Figure adapted from KEGG [60, 61]

Comparison of *C. jejuni* and *C. coli* identified 316 genes that are essential to both species (Fig. 3). There were 96 genes and 48 genes for *C. jejuni* and *C. coli*, respectively, that were not essential for the other species, despite some of these genes forming part of the core cross-species genome (Fig. 3, Table 5). For example, there were differences between the essentiality of genes belonging to the purine and pyrimidine biosynthesis pathways, with more genes being essential to *C. jejuni* then *C. coli* specifically in the purine pathway. Purines and pyrimidines are critical to both survival and replication of bacteria. The synthesis of nucleotides is essential for many cellular functions such as the synthesis of DNA, RNA, ATP, GTP and in metabolism. The de novo purine biosynthesis pathway produces inosine monophosphate which then can be made into guanosine triphosphate or adenosine triphosphate (Fig. 4). The de novo pyrimidine pathway produces cytosine, thymine, and uracil nucleotides (Fig. 4).

We identified multiple genes that belong to the purine pathway that were essential to *C. jejuni* but not for *C. coli.* These genes included *purS*, *purL, purE, purC, purH, purB, adk *and* guaB*. The proteins PurC/E/H/L/S form part of the pathway that biosynthesises inosine monophosphate, whilst GuaB synthesises guanosine monophosphate and PurB and Adk synthesise adenosine mono- and di-phosphate from inosine monophosphate in the branched pathways (Fig. 4). There were only two genes (*purQ* and *ndk*) that were essential to *C. coli*. Although *purQ* exists in all *C. jejuni* strains, in two strains (81–176 and M1cam) the *purQ* is non-functional and is annotated as a pseudogene due to a frameshift, so although the gene is called as essential to other *C. jejuni* strains, it does not form part of the core essential genome of *C. jejuni* and thus is only called essential in *C. coli*. The genes encoding PurF, PurM and PurA are essential to neither *C. jejuni* nor *C. coli*. PurF has been previously been described as essential to *C. jejuni* 81–176 [62], our findings corroborate this for that strain and two other strains (80512 and 80864). However, PurF was not essential in the other strains, thus it is defined as not essential to *C. jejuni*. The genes *purD*, *purB*, *guaB*, and *adk* were defined as essential for both species. Comparison to other TraDIS studies, where multiple strains and different Tns were also tested, using different media and atmospheric conditions to those used in this study, highlights that GuaB and Adk may be good targets for intervention as genes encoding these proteins were also essential in those studies [21, 23]. For the pyrimidine biosynthesis pathway, the following genes were not essential in either species, *carAB*, *pyrC*, *pyrD* and *pyrF*. The genes *pyrB* and *ndk* were essential only to *C. coli* and *pyrE* and *pyrH* were essential to *C. jejuni* (Fig. 4). The gene *pyrG* was the only member of the pyridimine biosynthesis pathway that was essential to both species. The genes *pyrH* and *pyrG* were found across different studies to be essential to *C. jejuni*, and therefore may be candidate targets for intervention [21, 23].

In *Staphylococcus aureus* and other bacterial species, there is an increasing link between nucleotide biosynthesis regulation and virulence despite the mechanisms not being completely understood [63]. A *Campylobacter* study using a Tn library in a mouse infection model found that inactivation of genes in the purine and pyrimidine biosynthesis pathways (*purS, purQ purL,carB, pyrB* and *pyrF)* resulted in severe colonization defects in mice [22]. These studies show that bacteria require nucleotide synthesis (or acquisition) to colonise a host, infect or replicate in the host. Therefore, the essential nature of these pathways may be of interest for therapeutic development. Unidentified purine and pyrimidine transporters have been suggested, but not found, in *Campylobacter*, as the rest of the purine salvage pathway is absent, apart from *apt* [22, 62]. In this study, the *apt* gene was not part of the core essential genome in either species. It is present in all *C. jejuni,* and *C. coli* strains tested; it was essential in all strains but one of the *C. jejuni* strains tested (80512) and only in one of the *C. coli* strains tested (15–537260).

Four aminoacyl tRNA synthetases appear in the essential gene list for *C. jejuni* only. As aminoacyl tRNA synthetases are normally essential, this warranted further investigation. For *C. coli* H102680185, *pheS*, *proS,* and *alaS* were found to have a very low number of Tn insertions (two, two and four Tn insertions, respectively) therefore missing the cut-off for being called as essential in that strain. All other strains had no insertions in these genes. Similarly, *gatB* had two Tn insertions found in *C. coli* 15–537360 thus missing the threshold for essentiality. None of the other *C. coli *strains had any Tn insertions in *gatB*.

Among the genes identified as being essential to just *C. coli* were those coding for a potassium uptake system (KtrA/B). Maintenance of osmotic pressure is essential to bacteria, which use potassium import systems to regulate this. Both this study and others have been unable to identify another potassium transporting system occasionally found in *C. jejuni* called KdpA/B/C, in the genomes for the *C. coli* strains used in this study or in other *C. coli* strains [22]. Thus, we hypothesise that *C. coli* contains only one potassium uptake system, encoded by *ktrA* and *ktrB*, which is, therefore, essential. Neither the genes encoding KtrA/B nor the genes encoding the KdpA/B/C system were identified as part of the core essential genome for *C. jejuni* in this study. This is due to the strain differences between *C. jejuni* strains. Within this study, *ktrA/B* are called as essential in three *C. jejuni* strains – 11168, 80512 and 80864, but are not essential in 81–176, M1cam and 50520408. In the strains where *ktrA/B* are not essential, the *kdpA/B/C* system exists. In *C. jejuni* 81–176 which has both systems, both have previously been found to be non-essential in *C. jejuni* 81–176 during in vitro growth [22, 64]. We hypothesise from these and others' results, that *Campylobacter* requires at least one functional potassium uptake system and strains that have both systems can rely on the other if one is removed, whereas for *Campylobacter* species such as *C. coli* with only one system, the genes encoding these proteins are essential.

These comparisons highlight the number of genes encoding hypothetical proteins and how much we do not yet understand, even in the most well-studied *Campylobacter* genomes. Many genes encoding hypothetical proteins have been found to be essential but have no assigned function. There are also genes encoding proteins with unknown functions but assigned to be a membrane protein. Proteins that have a membrane or periplasmic location, such as lipoproteins, are of particular interest due to the fact they are on the surface of the bacteria and therefore present accessible targets for intervention [65]. Within the *C. jejuni* essential genes, 12 of these 96 encode hypothetical proteins or encode a predicted membrane associated protein (such as membrane protein/periplasmic protein or lipoprotein but are not assigned a function) (Fig. 3A). All but one of these proteins form part of the core genome between the *C. jejuni* and *C. coli* strains used in this study*.* Periplasmic protein Cj0965 (locus tags from *C. jejuni* 11168) was not found in *C. coli* but is essential to *C. jejuni.* There is little known about Cj0965, but it has been found in outer membrane and periplasmic fractions of *C. jejuni* 11168H [66]. A scan of the InterPro database predicts a signal peptide region, followed by a region of membrane protein that would be outside the membrane in the extracellular region [67].

From the eleven hypothetical or membrane associated genes that are essential to *C. jejuni* but are part of the core genome between *C. jejuni* and *C. coli,* five are annotated as being periplasmic or membrane proteins. Two adjacent genes, Cj0151c and Cj0152c (periplasmic and membrane protein, respectively) are in this list and interestingly, Cj0152c has been previously identified as an immunogenic protein, recognised by rabbit anti-*C. jejuni* antisera raised against whole cells of *C. jejuni* ATCC29428 [68]. One of the hypothetical proteins, Cj0331c is encoded by one of the 49 genes that form a molecular signature of epsilon proteobacteria and form part of an oxygen response signature seen in *Campylobacter* [69, 70].

In *C. coli,* two of the 48 essential genes encode hypothetical proteins. Both are also present in *C.jejuni* and therefore are not species-specific targets. These were N149_1044 and N149_01895 (locus tags from *C. coli* 15–537360). Little is known about either of these proteins. InterPro searches returned no insight for N149_1044 but for N149_01895 there was a predicted signal peptide followed by a membrane bound protein with a region outside the membrane in the extracellular region.

The addition of the data from the Tn libraries created in *C. lari* 35221 and *C. hyointestinalis* 35217 enabled us to perform a comparison between all four species (Fig. 3b, Table 5). We identified 977 genes as homologs across all four species and 261 genes essential to all four species (reduced from 316 genes that were core and essential to both *C. jejuni* and *C. coli*). Sixteen genes were essential to only *C. jejuni* and *C. coli* (Fig. 3b), despite at least six of these belonging to the core genome from all four species. The six genes that were identified as being core across all species, but only essential to *C. jejuni* and *C. coli,* encode the following: a hypothetical protein (Cj0797c), alanine racemase (Cj0905c), a periplasmic protein with a murein L,D-transpeptidase region (Cj0906c), the major outer membrane protein PorA (Cj1259), sulfate adenyltransferase (Cj1609), and a Na^+^/H^+^ antiporter (Cj1655c). Four of these proteins are associated with the cell wall, and therefore may be interesting therapeutic targets. PorA is the major outer membrane protein that has been shown to have a role in adherence and has previously been investigated as an immunogenic antigen [71, 72]. It has also been suggested as a core virulence gene in a study using 39 representative *Campylobacter* genomes. Interestingly, here it is core across all strains tested but is only essential in *C. jejuni* and *C. coli.* Alanine racemase catalyses the conversion of L-Alanine to D-Alanine which forms part of the cell wall peptidoglycan [71], whilst murein L,D-transpeptidase is a peptidoglycan crosslinking enzyme that contributes to the synthesis of peptidoglycan. The Na^+^/H^+^ antiporter is one of two Na^+^/H^+^ antiporters present within *C. jejuni* and *C. coli* and has an essential role in maintaining pH levels. It is unclear if the two require each other or act in tandem, but Cj1655c could complement a growth deficiency in *E. coli* cation/proton antiporter mutant, suggesting it can function alone [73]. The non-membrane associated proteins essential to *C. coli* and *C. jejuni* included a gene (Cj0797c) encoding a hypothetical protein whose functional domain matches to a Phd/YefM antitoxin protein family and another gene (Cj1609) encoding a sulfate adenyltransferase, which is an enzyme that is involved in purine metabolism and sulphur metabolism. Three of the sixteen genes essential to both *C. jejuni* and *C. coli* are present but not essential to *C. lari* 35221. These were *thiS,* encoding a sulfur biosynthesis protein, Cj0682, encoding a hypothetical protein with no functional domains predicted and Cj1575c, annotated as encoding a hypothetical protein but predicted to act as an electron acceptor, alongside Cj1574c [74]. The remaining seven essential *C. jejuni* and *C. coli* genes were only present in these two species. Four of these genes encode for hypothetical proteins. One of the hypothetical proteins, Cj0851c, is predicted to be a membrane protein and the other, Cj0135 is predicted to have a YlxR domain. For both of the other hypothetical proteins, Cj0839c and Cj0395c, Interpro predicted no known functional domains. The other core essential genes to *C. jejuni* and *C. coli* were two genes that encode membrane associated proteins – CorA (magnesium/cobalt transporter), and TatA (which forms part of the twin-arginine translocase secretion system). *C. jejuni* 11168 has been found to have two paralogues of TatA that exist in all *C. jejuni* strains but the paralogue only exists in one strain of *C. coli* used in this study [70]. We identified *tatA1* (Cj1176c) as essential. TatA1 is responsible for the correct periplasmic activity of sulphite oxidase, copper oxidase and alkaline phosphatase and it has been suggested that TatA2 is unable to function correctly in the absence of TatA1 [75].The other essential gene for *C. jejuni* and *C. coli* that was identified was the 50S ribosomal protein L34.

There were 261 essential genes that were common to all four species of *Campylobacter* (Fig. 3b, Tables 5 and 6). Perhaps unsurprisingly, many of the 261 genes encode proteins that have been found before to be essential to other bacteria. For example, *rpoD,* encoding the housekeeping sigma factor 70, genes such as *dnaA* and *dnaN,* and many genes encoding ribosomal proteins were identified as essential in all *Campylobacter* strains and species tested. There have been several studies that have looked at the core genome across *Campylobacter* species. As our study looks at core genes of a smaller subset of strains and species but also looks at essentiality it is interesting to compare between these studies. The gene *htrB* is essential across all strains and species tested in this study. HtrB encodes a lipid A biosynthesis lauroyl acyltransferase, and is involved in lipid A biosynthesis, which with the core oligosaccharide forms the LPS. Previous attempts to delete *htrB* in *C. jejuni* have failed, further supporting its essentiality [76]. Zhong et al. interestingly highlighted the gene *htrB* as a core virulence gene across 39 representative *Campylobacter* species [77]. The other core virulence genes that they found were *porA, PEB4, cheY, Cj1135* and *kspF.* As previously mentioned, *porA* formed part of the core genome but was only essential in two species in our study. PEB4 (Cj0596) is core and essential across all strains within this study. The gene *PEB4* encodes apeptidyl prolyl *cis–trans* isomerase located within the periplasm. Gene deletion mutants can be made however in these mutants, where the outer membrane protein profile changes, resulting in a change in virulence and an increased susceptibility to antimicrobials [78, 79]. The gene Cj1135 is part of the LPS biosynthesis cluster, and does not form part of the core genome across the six strains of C. *jejuni* as the gene is not present in *C. jejuni* 50520408. In *C. coli*, Cj1135 (N149_1076) is core across all four strains but is not essential to all the strains. The gene *kpsF,* encoding a a D-arabinose 5-phosphate isomerase, is core to all strains and species in this study but only essential to five of the *C. jejuni* strains and three of the *C. coli* strains. The gene *cheY,* encoding a chemotaxis protein, is core to all strains and species in this study, however, it is not essential, most likely to reasons explained previously, regarding motility and a lack of need under these rich conditions. Another study used 173 *C. jejuni* strains and identified five core virulence factors with high antigenicity as potential core vaccine targets. These were *sodB, htrA, cadF, flgC, kpsD* [80]. All genes were core to all strains and species used in this study, except for *kpsD* which was not found in *C. hyointestinalis* 35217. None of the genes were found in the core essential genes for *C. jejuni* and *C. coli* nor the essential gene lists for *C. lari or C. hyointestinalis*, except for *sodB* which was essential for *C. lari* 35221. Within the list of 261 core essential genes across the four species, there were genes that encode proteins that have previously been considered and investigated as possible targets for intervention in other bacteria, such as the highly conserved Obg GTPase [81]. Another protein, Cj0404, has previously been tested as a potential vaccine candidate, whilst Cj1090c, a lipoprotein, which has recently been shown to be LptE [82] has been used as a target in a phage-display screening campaign [83, 84]

**Table 6:**
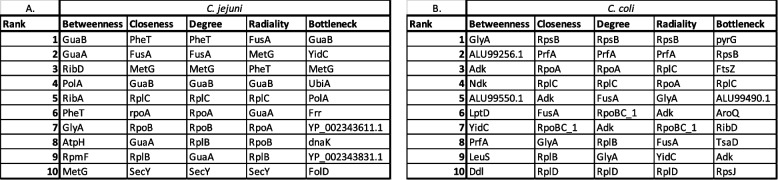
Predicted hub proteins from the essential gene lists for *C. jejuni* (A) and *C. coli* (B). *C. jejuni* and *C. coli* top 10 hub proteins were predicted using Betweenness, Closeness, Degree, Radiality and Bottleneck to rank nodes within the protein-protein interaction networks built from the essential gene lists. The top 10 proteins are in rank order (1 being the highest and 10 being the lowest) according to species and the type of analysis

Included in the essential gene list across all four species are 21 genes of unknown function, with eight of these predicted, by Bakta, to be a periplasmic proteins or integral membrane proteins. These eight include two which have been experimentally shown to be *N*-glycosylated (Cj0515 and Cj0503) [85], four which have been found to have functions, but have not been annotated as such (Cj1276c – FtsX, Cj0801 – MurJ, Cj0362 – HemJ and Cj0313 – LptF/G). Two genes, Cj0651 and Cj1637c, have no functional domains predicted but are annotated as an integral membrane protein and a periplasmic domain respectively.

We performed a BlastP search against non-redundant protein sequences excluding those from *Campylobacter.* For Cj0651 we were unable to find any proteins with similar sequence in any other bacteria outside the Campylobacterales order. This protein warrants further investigation as a possible Campylobacterales-specific, core-essential, surface-accessible target. From the rest of the hypothetical genes that were not annotated as being membrane associated, two genes have previously been highlighted in previous essential gene lists. The genes, Cj0711 and Cj1712, have been found to be essential across multiple Tn mutagenesis studies. Interestingly Cj1712 has been found to encode a putative PunB homolog thus likely be involved in purine and pyrimidine metabolism [21].

### Network topology analysis of core essential genes

Using the essential gene lists, it is possible to explore the interactions of proteins, encoded by these genes, using protein–protein interaction (PPI) networks. Here we have used the essential gene list to visualize the interactions of essential proteins within *Campylobacter,* with each node representing a protein connected by edges representing interaction between the proteins. The PPI network was then analysed for ‘Hub’ proteins. Hub proteins are of a particular interest as these are proteins that have a high number of interaction partners across the protein network. Due to their vast number of interactors and connectivity, which indicates importance of that protein, targeting these proteins may offer advantages for intervention. When eliminating or targeting hub proteins, the entire structure of a biological network changes, and can have an impact on fitness [86].

Protein–protein interaction networks were created with the list of essential genes that we identified as being included in the core genome of *C. jejuni* and *C. coli* strains tested in this study. These PPI networks were created by STRING and visualised using Cytohubba [49]. For the STRING analysis, the network was built using *C. jejuni* 11168 as the representative strain for *C. jejuni.* For *C. coli,* none of the strains used in this study were represented within the STRING database, and so homologs of the essential genes were identified in *C. coli* NCTC11366, the only clade 1 *C. coli* in the STRING database.

The PPI network for *C. jejuni* comprises 412 proteins (or nodes) with 8834 interactions (edges) whereas for *C. coli* the PPI network comprises 338 proteins (nodes) with 7187 interactions (edges). Centrality analysis was conducted on these networks to integrate the proteins and their functional relevance within the network. Centrality analysis was conducted within Cytohubba and the top 10 potential hub proteins were predicted using several topological network analysis methods used for predicting hub proteins – Degree [87], Betweenness [88], Closeness [89], and Radiality [90] and Bottleneck [91]. Previous studies in *Helicobacter pylori* have combined all five methods to find overlapping hub proteins [48]. Using this approach, two proteins were identified across five methods in the *C. jejuni* core essential network – these were MetG (Cj0838c) and GuaB (Cj1058c). In *C. coli*, only one protein was identified, this was Adk (N149_0609).

Interestingly, proteins within the purine pathway were identified as potential hub proteins for both *C. jejuni* and *C. coli.* GuaB (*C. jejuni*) is part of the guanine biosynthesis pathway, Adk (*C. coli*) is part of the adenine biosynthesis pathway. Intriguingly, purine pathway members have been identified as main network hub nodes in the closely related bacterial species *Helicobacter pylori* 26695 [48], suggesting that these might be good targets for intervention for these important gastrointestinal pathogens.

## Conclusion

In this study, a combination of core genome analysis, the use of the molecular technique Tn mutagenesis, and bioinformatic analysis pipelines were employed to identify the core essential genes within and across *Campylobacter* species. Overall, we identified 412 core essential genes within *C. jejuni* and 364 core essential genes within *C. coli*. Comparison between *C. jejuni* and *C. coli,* revealed 316 core essential genes (*i.e*., core and essential in all *C. jejuni* and *C. coli* strains tested), and when adding in one Tn mutant library from both *C. lari* and *C. hyointestinalis*, this reduced to 261. This type of analysis provides the opportunity to identify *Campylobacter-*specific targets that are essential either in a species-specific manner or across/between species. Interestingly, *htrB* and *PEB4* which have been found to be core virulence genes across *Campylobacter*  spp. are essential and therefore would warrant further investigation [77]. The core essential gene list produced indicates that there are many essential genes that are critical to the fitness and/or survival of *Campylobacter* but have a hypothetical/unknown function. Characterization of these genes and their translation products will provide vital insights into the fundamental biology of *Campylobacter* and enable a rational approach to the development of novel interventions. With these conserved and essential hypothetical genes, it will be useful to combine additional genomic methods with other molecular approaches (*e.g.,* CRISPRi, TraDIS-Xpress), for genotype-to-phenotype analysis, to better understand the function of these genes.

## Supplementary Information

**Additional file 1:  Table S1.** Campylobacter species and strains tested for Tn mutagenesis.

**Additional file 2: Table S2.** Customized sequencing primers. Nextera adapter i5 (underlined/green), Index (black), Read 1 sequencing primer (dashed line, brown), spacer (lowercase, black), binding site for transposon (bold underlined, purple). 

**Additional file 3: Table S3. **This excel spreadsheet contains essential gene lists for *C.jejuni *strains (Sheet 1), *C. coli* strains (Sheet 2), *C. hyointestinalis* (Sheet3) and *C. lari* (Sheet 4)

**Additional file 4: Table S4. **Contains the output from Panaroo for *C. jejuni*, the filtered output, the output from Panaroo for *C. coli*, the filtered output and the core and essential gene list across *C. jejuni* and *C. coli*.

**Additional file 5: Table S5. **Output from core genome analysis across species.

**Additional file 6: Figure S1. **Gamma fitted distribution plots created by the Bio-Tradis software.

## Data Availability

The data that support the findings of this study are openly available in EBI ArrayExpress at https://www.ebi.ac.uk/biostudies/arrayexpress/studies/E-MTAB-12314, reference number E-MTAB-12314.
