# Supplementary material for: Application of TraDIS to define the core essential genome of Campylobacter jejuni and Campylobacter coli
Source: BMC Microbiol. 2023 Apr 6;23:97. doi: 10.1186/s12866-023-02835-8 (PMC10077673; doi:10.1186/s12866-023-02835-8)
Supplement: Supplementary file 6 — Additional file 6: Figure S1. Gamma fitted distribution plots created by the Bio-Tradis software. [file 12866_2023_2835_MOESM6_ESM.pdf]

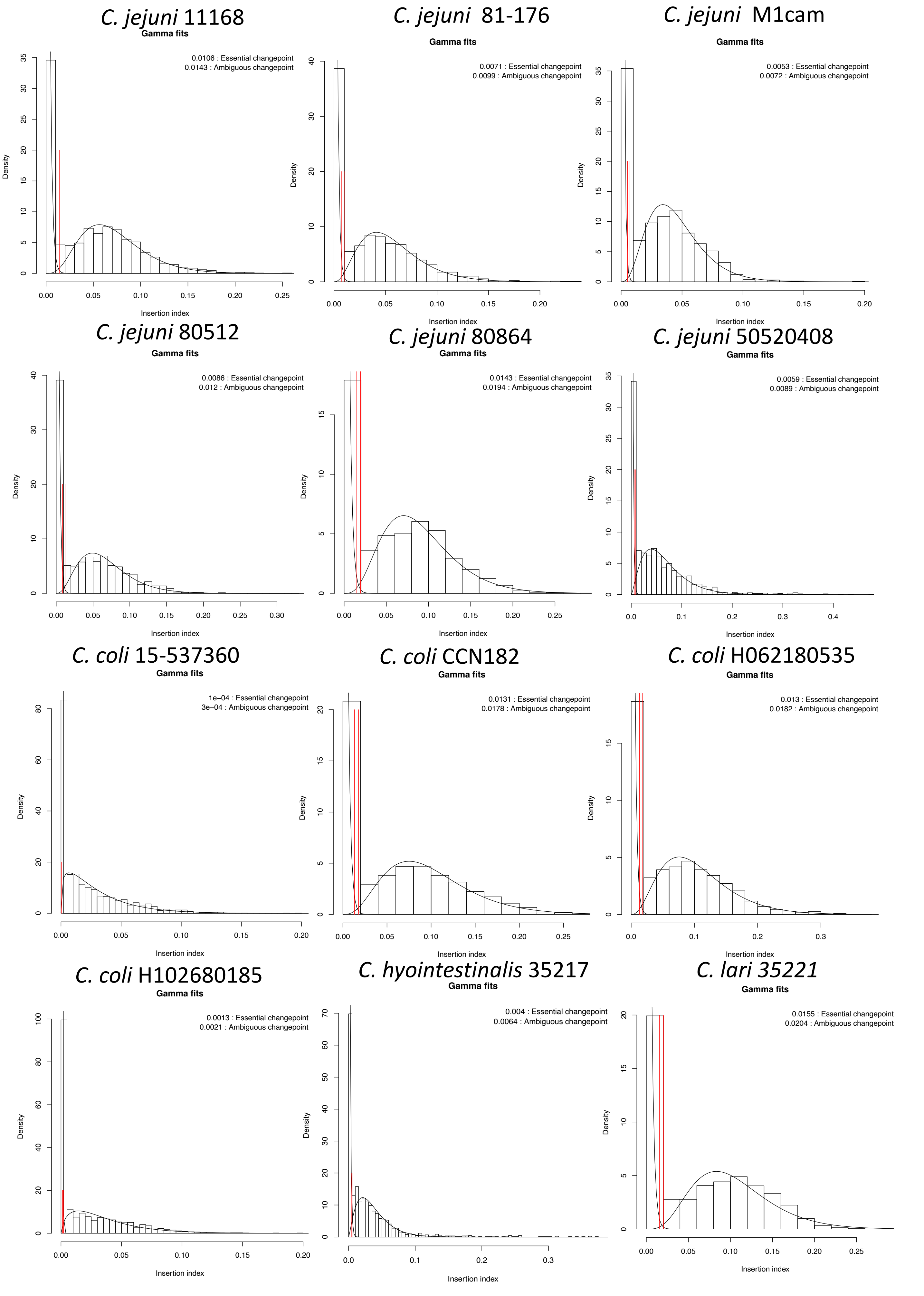

C. jejuni 11168
Gamma fits
0.0106 : Essential changepoint
0.0143 : Ambiguous changepoint
Density
Insertion index
C. jejuni 81-176
Gamma fits
0.0071 : Essential changepoint
0.0099 : Ambiguous changepoint
Density
Insertion index
C. jejuni M1cam
Gamma fits
0.0053 : Essential changepoint
0.0072 : Ambiguous changepoint
Density
Insertion index
C. jejuni 80512
Gamma fits
0.0086 : Essential changepoint
0.012 : Ambiguous changepoint
Density
Insertion index
C. jejuni 80864
Gamma fits
0.0143 : Essential changepoint
0.0194 : Ambiguous changepoint
Density
Insertion index
C. jejuni 50520408
Gamma fits
0.0059 : Essential changepoint
0.0089 : Ambiguous changepoint
Density
Insertion index
C. coli 15-537360
Gamma fits
1e−04 : Essential changepoint
3e−04 : Ambiguous changepoint
Density
Insertion index
C. coli CCN182
Gamma fits
0.0131 : Essential changepoint
0.0178 : Ambiguous changepoint
Density
Insertion index
C. coli H062180535
Gamma fits
0.013 : Essential changepoint
0.0182 : Ambiguous changepoint
Density
Insertion index
C. coli H102680185
Gamma fits
0.0013 : Essential changepoint
0.0021 : Ambiguous changepoint
Density
Insertion index
C. hyointestinalis 35217
Gamma fits
0.004 : Essential changepoint
0.0064 : Ambiguous changepoint
Density
Insertion index
C. lari 35221
Gamma fits
0.0155 : Essential changepoint
0.0204 : Ambiguous changepoint
Density
Insertion index

S1 Figure: Gamma fitted distribution plots created by the Bio-Tradis software. For each bar chart, the number of transposon insertions in each gene is divided by the gene length to give the “insertion index”, and this value is plotted along the x-axis. The y-axis indicates the frequency of genes that fall within short ranges of insertion index values. The bimodal distribution shows the essential genes at or near x=0 (few or no insertions per gene length) and the non-essential genes further along the x-axis (numerous insertions per gene length). The gamma fit (line) indicates the trough-minimum between the two modes, providing an appropriate insertion index to distinguish between essential and non-essential genes. The recommended Bio-Tradis insertion index to make this distinction is shown in the top right of each chart.
